# An eye-tracking study of interpersonal threat sensitivity and adverse childhood experiences in borderline personality disorder

**DOI:** 10.1186/s40479-020-00141-7

**Published:** 2021-01-04

**Authors:** Katja I. Seitz, Johanna Leitenstorfer, Marlene Krauch, Karen Hillmann, Sabrina Boll, Kai Ueltzhoeffer, Corinne Neukel, Nikolaus Kleindienst, Sabine C. Herpertz, Katja Bertsch

**Affiliations:** 1 Department of General Psychiatry, Center for Psychosocial Medicine, Medical Faculty, Heidelberg University, Heidelberg, Germany; 2 Institute for Psychiatric and Psychosomatic Psychotherapy, Central Institute of Mental Health, Medical Faculty Mannheim, Heidelberg University, Mannheim, Germany; 3 Department of Psychology, Ludwig-Maximilians-University Munich, Munich, Germany

**Keywords:** Adverse childhood experiences, Borderline personality disorder, Childhood maltreatment, Eye-tracking, Emotion dysregulation, Facial affect recognition, Interpersonal functioning, Threat hypersensitivity

## Abstract

**Background:**

Previous eye-tracking studies provide preliminary evidence for a hypersensitivity to negative, potentially threatening interpersonal cues in borderline personality disorder (BPD). From an etiological point of view, such interpersonal threat hypersensitivity might be explained by a biological vulnerability along with a history of early life adversities. The objective of the current study was to investigate interpersonal threat hypersensitivity and its association with adverse childhood experiences (ACE) in patients with BPD employing eye-tracking technology.

**Methods:**

We examined a sample of 46 unmedicated, adult female patients with BPD and 25 healthy female volunteers, matched on age and intelligence, with a well-established emotion classification paradigm with angry, fearful, happy, and neutral facial expressions. ACE were assessed retrospectively with the Childhood Trauma Questionnaire.

**Results:**

Patients as compared to healthy volunteers reflexively directed their gaze more quickly towards the eyes of emotional and neutral faces and did not adapt their fixation patterns according to the facial expression presented. Misclassifying emotional and neutral faces as angry correlated positively with the patients’ self-reported ACE.

**Conclusions:**

Building on and extending earlier findings, our results are likely to suggest a visual hypervigilance towards the eyes of emotional and neutral facial expressions and a childhood trauma-related anger bias in patients with BPD. Given the lack of a clinical control group, the question whether these findings are specific for BPD has to remain open. Thus, further research is needed to elucidate the specificity of altered visual attention allocation and the role of ACE in anger recognition in patients with BPD.

## Background

Borderline personality disorder (BPD) is characterized by persistent and profound emotion dysregulation [1]. Emotion dysregulation in BPD encompasses emotion sensitivity, heightened and labile negative affect, deficient appropriate and excessive maladaptive emotion regulation strategies according to Linehan’s biosocial model [2]. Emotion sensitivity, specifically the sensitivity to negative or potentially threatening interpersonal signals, has been addressed in a number of recent review articles [3–7]. Despite some inconsistencies (cf. [8, 9]), there is considerable empirical support for the hypothesized hypersensitivity to interpersonal threat cues in BPD [3]. Experimental findings in mostly female patients with BPD indicate a negatively biased perception of neutral, ambiguous, or positive facial expressions (“negativity bias”; [10–12]), particularly a higher sensitivity in recognizing anger in other emotions (“anger bias”; [13–17]) [but, for inconsistent results, see [18, 19].

Adverse childhood experiences (ACE) are seen as playing a significant role in the etiology of interpersonal threat hypersensitivity in BPD. Patients with BPD report high rates of ACE such as emotional neglect, physical maltreatment, or sexual abuse [20, 21]. Following Linehan’s biosocial model [22], interpersonal threat hypersensitivity in BPD may emerge from an interplay between biological vulnerabilities and the exposure to such traumatic childhood experiences [1]. Studies suggest that the more patients with BPD report ACE, the more they show a tendency to judge faces as less approachable [23], a heightened vigilance [24], and avoidant reactions towards angry faces [25], but the evidence remains somewhat inconclusive (cf. [17, 26]).

To disentangle previous inconsistent findings, three recent studies have implemented eye-tracking technology to capture ecologically more valid information about the attentional mechanisms relevant for perceiving facial expressions in patients with BPD [27–29]. Kaiser et al. [29] found female patients with BPD to be more likely to characterize ambivalent emotional blends as angry compared to a clinical control group (Cluster-C personality disorder) and to show prolonged fixations on the eye region of ambiguous facial stimuli compared to a non-patient control group. The eye region is known to convey the most crucial information about threat-related facial expressions [30, 31]. Thus, fixating extensively on the eyes of emotional faces might indicate a hypervigilance towards interpersonally relevant and potentially threatening cues, irrespective of the faces’ emotional valence [32]. Interestingly, in the study of Kaiser et al. [29] the attentional bias towards the eyes of ambiguous facial expressions was found to be most pronounced in patients with BPD and comorbid posttraumatic stress disorder (PTSD), suggesting a significant role of traumatic experiences. In two own studies, we also revealed a biased visual attention in female patients with BPD towards the eyes of emotional facial expressions displayed in full intensity [27, 28]. In a functional neuroimaging study, patients with BPD exhibited more and faster initial saccades towards the eyes of angry faces compared to a healthy control group. This interpersonal threat hypersensitivity was associated with increased amygdala activation [28]. In a consecutive small behavioral study, patients with BPD showed a tendency to misclassify emotional and neutral faces as angry, slower saccades away from the eyes of fearful faces and faster saccades towards the eyes of neutral faces compared to healthy volunteers [27]. So far, only one eye-tracking study in patients with BPD has investigated the association between interpersonal threat hypersensitivity and traumatization [29]. Since the authors did not analyze the association of ACE and indicators of interpersonal threat hypersensitivity, the question whether the anger bias and hypervigilance towards the eyes of emotional faces is indeed associated with early life adversity remains open.

The aim of the present study was to investigate the association of ACE with the proposed interpersonal threat hypersensitivity in patients with BPD employing eye-tracking technology. We hypothesized to find a biased perception of interpersonal threat cues in patients with BPD compared to healthy volunteers in terms of (1) more misclassifications of facial expressions as angry, (2) more and faster initial saccades towards the eyes of angry faces, and (3) longer fixation durations on the eye region regardless of the emotion the faces are displaying. Moreover, we hypothesized that (4) the overall severity of self-reported ACE would correlate positively with indicators of threat hypersensitivity.

## Methods

### Participants

In total, 46 unmedicated female patients with a current DSM-IV diagnosis of BPD (BPD, *M*_age_ = 28.6, *SD* = 7.5, range = 18–46 years) and 25 healthy women who had never received a psychiatric diagnosis or undergone any psychotherapeutic or psychiatric treatment (CON, *M*_age_ = 26.4, *SD* = 5.5, range = 18–40 years) were included in the current analyses. The sample size is large enough to detect small group by condition interactions (η^2^ = .05) as well as medium to large correlations between self-report, behavioral, and eye-tracking data (*r* = .40) within the BPD sample with a statistical power of 1-β ≥ .80 [33]. Effect sizes of η^2^ ≥ .05 and correlations of *r* ≥ .40 may be expected according to previously published experimental behavioral and eye-tracking studies with female patients with BPD or major depression and healthy volunteers [23, 27, 28, 34].

Exclusion criteria for all participants comprised neurological disorders; current alcohol/drug abuse (urine toxicology screening) or alcohol/drug abuse in the last 2 months (interview); severe medical illness; use of psychotropic medication for at least 2 weeks before participation in the study; or impaired vision. Additional exclusion criteria for the patient sample were lifetime diagnoses of schizophrenia, schizoaffective or bipolar disorder and self-reported alcohol/drug dependence in the last 12 months. Initially, 56 patients and 28 healthy volunteers participated in the emotion classification task and filled out the Childhood Trauma Questionnaire (CTQ; [35]). Overall, 10 patients and 3 healthy volunteers had to be excluded due to equipment malfunction (patients: *n* = 8, healthy volunteers: *n* = 3), positive toxicology screenings (patients: *n* = 1) or neurological abnormalities, identified in a corresponding MRI study (patients: *n* = 1). The groups were matched with regard to age and intelligence (see Table 1 for detailed demographic and psychometric information and group comparisons).

**Table 1:** Demographic and psychometric information (mean [M] ± one standard deviation [SD]) of patients with borderline personality disorder (BPD) and healthy volunteers (CON)

	BPD (***n*** = 46)	CON (***n*** = 25)	Group comparison	
	***M ± SD***	***M ± SD***	***t*** or ***U***	***p***
Age (years)	28.6 ± 7.5	26.4 ± 5.5	1.27	.209
Intelligence (RAVEN)	112.0 ± 11.1	113.9 ± 9.7	−0.70	.486
Adverse Childhood Experiences (CTQ)				
Sum Score^a^	61.4 ± 19.4	32.0 ± 7.8	96.50	< .001
Emotional Abuse^a^	17.2 ± 5.5	7.4 ± 3.3	95.00	< .001
Physical Abuse	8.3 ± 4.5	5.6 ± 1.4	266.50	< .001
Sexual Abuse	9.1 ± 6.5	5.1 ± 0.3	300.00	< .001
Emotional Neglect	16.7 ± 6.4	8.0 ± 3.1	162.50	< .001
Physical Neglect	9.6 ± 3.6	5.9 ± 1.7	199.50	< .001
Borderline Symptomatology (BSL-23)^b^	1.6 ± 0.8	0.2 ± 0.3	37.00	< .001
Depressiveness (BDI-II)^b, c^	23.4 ± 10.6	3.0 ± 5.0	39.50	< .001
Trait Anxiety (STAI)^b^	62.1 ± 7.4	33.2 ± 8.2	15.03	< .001
Emotional Dysregulation (DERS)^a^	128.5 ± 20.0	62.8 ± 16.2	14.04	< .001
Current Comorbid Axis I Disorders (lifetime)				
Affective Disorders	18 (36)	0 (0)		
Posttraumatic Stress Disorder	8 (13)	0 (0)		
Other Anxiety Disorders	25 (29)	0 (0)		
Substance Use Disorders	0 (13)	0 (0)		
Eating Disorders	12 (24)	0 (0)		
Somatization Disorders (current only)	2	0		
Adjustment Disorder	0 (0)	0 (0)		
Current Comorbid Axis II Disorders (lifetime)				
Avoidant Personality Disorder	13 (13)	0 (0)		
Antisocial Personality Disorder	2 (2)	0 (0)		

Groups were matched with respect to age (in years) and intelligence (IQ scores). *RAVEN* Raven’s Standard Progressive Matrices, *CTQ* Childhood Trauma Questionnaire, *BSL-23* Borderline Symptom List (Short Version), *BDI-II* Beck Depression Inventory-II, *STAI* State-Trait Anxiety Inventory, *DERS* Difficulties in Emotion Regulation Scale

^a^ Data of one patient with BPD is missing . ^b^ Data of two patients with BPD are missing . ^c^ Data of one healthy volunteer is missing

Participants were recruited by the central project of the KFO-256, a Clinical Research Unit funded by the German Research Foundation, investigating mechanisms of disturbed emotion processing in BPD [36]. All projects from this research unit include participants from a joint database. Ethics approval was provided by the Ethics Committee of the Medical Faculty of the University of Heidelberg. All participants gave their written informed consent and were reimbursed for their participation.

### Measures

Diagnoses of BPD and co-occurring axis I and II disorders were assessed by qualified diagnosticians using the Structured Clinical Interview for DSM-IV (SCID-I for axis I diagnoses; [37]) and the International Personality Disorder Examination for DSM-IV (IPDE for BPD and comorbid avoidant and antisocial personality disorders; [38]). Prior to the study, diagnosticians completed a standardized diagnostic training, leading to high levels of inter-rater reliability (*ICC* ≥ .911).

ACE were measured with the Childhood Trauma Questionnaire (CTQ; [35]), assessing emotional, physical and sexual abuse, and emotional and physical neglect. BPD symptom severity (short version of the Borderline Symptom List, BSL-23; [39]), depressiveness (revised version of Beck’s Depression Inventory, BDI-II; [40]), trait anxiety (State-Trait Anxiety Inventory, STAI; [41]) and emotion regulation (Difficulties in Emotion Regulation Scale, DERS; [42]) were assessed with self-report questionnaires for further dimensional characterization of the present sample. Intelligence was assessed using Raven’s Standard Progressive Matrices [43] (see online supplement for psychometric properties of questionnaires).

As shown in Table 1, patients with BPD revealed higher scores in all state and trait questionnaires compared to healthy volunteers, with the exception of Raven’s Standard Progressive Matrices (matching variable). Patients with BPD reported more severe ACE, more BPD symptoms, and higher levels of emotion dysregulation, depressiveness, and trait anxiety.

### Emotion classification paradigm

The emotion classification task was based on a 2 × 2 × 4-design, with the within-subject factors presentation time (150 ms, 5000 ms), initial fixation (eyes, mouth) and emotional expression (angry, fearful, happy, neutral) (for similar designs, see [27, 32, 44, 45]). Employing these two presentation time conditions allows for investigating reflexive attentional shifts (150 ms) as well as sustained attention (5000 ms) towards diagnostically relevant facial features. In each presentation time condition, participants were required to classify 80 emotional faces, unambiguously displaying angry, fearful, happy, and neutral expressions (see also online supplement), as quickly and as accurately as possible by pressing one of four corresponding keys. To control for initial fixation, half of the faces within each emotional category were unpredictably shifted either downward or upward in each trial. Each trial started with the presentation of a fixation cross (2000 ms), followed by a facial stimulus with the eyes or the mouth appearing at the former position of the fixation cross (brief condition: 150 ms; long condition: 5000 ms), and a blank screen in the brief condition only (1850 ms). Each trial ended with another fixation cross with a variable duration (1000–3000 ms) to reduce anticipation effects.

### Experimental protocol and data acquisition

All participants underwent a telephone screening for inclusion and exclusion criteria (approximately 45 min), and an onsite diagnostic session (approximately 3 h). After excluding acute substance abuse by a urine toxicology screening, participants performed the emotion classification task (approximately 1 h, including instruction, training, and short breaks between presentation time blocks).

Eye-tracking data were recorded with a 60-Hz monocular eye-tracking system (ViewPoint, Arrington Research, Scottsdale, AZ, USA). The head location was fixed using a chin rest and a forehead bar with a viewing distance of 57 cm. Before each experimental block, the eye-tracking camera was adjusted, eight training trials were presented and nine-point calibration was applied. Stimulus presentation and response recording were performed using the software Presentation (Version 16.3, Neurobehavioral Systems, Albany, CA, USA). Stimuli were presented on an Eizo FlexScan S2202 display (47.5 cm × 30.0 cm) with a resolution of 1680 × 1050 pixels and a refresh rate of 60 Hz.

### Data analysis

Details are presented in the online supplement. Following the procedure of previous studies (e.g., [27, 32]), we extracted behavioral data (i.e., proportion of correct responses, types of errors, response latencies in trials with correct emotion classifications) and eye-tracking data (i.e., proportion and latency of initial saccades, fixation durations). Initial saccades were classified according to whether they were directed towards the other major facial feature (i.e., eyes, mouth). When the eyes were presented at the former position of the fixation cross, the proportion and latency of the downward fixation changes towards the mouth were scored, whereas when the mouth was shown at fixation, the proportion and latency of the upward fixation changes towards the eyes were scored. Fixation durations were defined as the cumulative amount of time participants spent looking at either the eye or the mouth region in the long condition. Proportion of correct responses, response latencies as well as proportion and latency of initial saccades were submitted to 2 × 2 × 2 × 4 mixed-design analyses of variance (ANOVAs) with the between-subject factor group (BPD, CON) and the within-subject factors presentation time (brief, long), initial fixation (eyes, mouth), and emotional expression (angry, fearful, happy, neutral). Types of errors were analyzed using a 2 × 2 × 4 mixed-design ANOVA with the between-subject factor group (BPD, CON) and the within-subject factors presentation time (brief, long) and error type (misclassifications of faces as angry, fearful, happy, neutral). Finally, fixation duration data (only available in the long presentation time condition) were subjected to a 2 × 2 × 4 mixed-design ANOVA with the between-subject factor group (BPD, CON) and the within-subject factors facial feature (eyes, mouth) and emotional expression (angry, fearful, happy, neutral). In accordance with previous studies with the same experimental paradigm (e.g., [27, 43]), ANOVAs were also conducted separately for both presentation time conditions to identify additional group effects. We applied the Huynh-Feldt procedure [46] to correct for potential violations of the sphericity assumption where indicated. Partial eta squared (η^2^) is provided as an effect size index. In cases of significant effects, Dunn’s Multiple Comparisons are reported as post-hoc tests.

Furthermore, we performed bivariate correlational analyses between self-reported ACE of patients with BPD (CTQ sum score), and behavioral (i.e., misclassifications as angry) and eye-tracking measures of interpersonal threat hypersensitivity (i.e., proportion and latency of initial saccades towards the eyes or the mouth of angry faces, fixation durations on the eyes or the mouth of angry faces). Correlations are reported as Pearson product-moment correlation coefficients (*r*) with Bonferroni-Holm correction for multiple comparisons [47].

Statistical analyses were conducted using the statistical programming language R (version 3.6.2) and SPSS for Windows (version 26). A significance threshold of *p* < .05, two-tailed, was set for all statistical analyses.

## Results

### Hypothesis 1

**Fig. 1:**
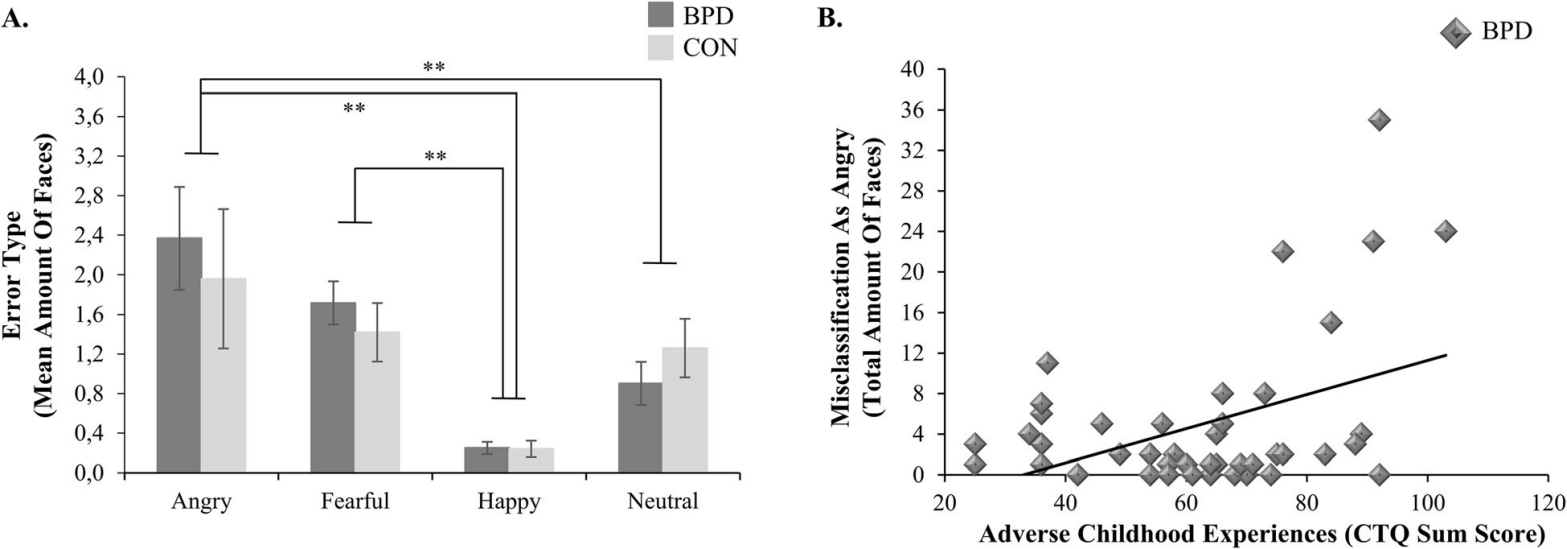
Anger bias in patients with BPD and healthy volunteers and its association with patients’ self-reported ACE. **a**. Types of errors (mean amount of errors ± one standard error) of patients with borderline personality disorder (BPD) and healthy volunteers (CON) across presentation time conditions. The displayed graph represents the significant main effect of error type, with significantly more misclassifications as angry than as happy or as neutral and significantly more misclassifications as fearful than as happy in both groups. Groups did not differ in their types of errors. ** *p* < .01. **b**. Association of patients’ self-reported ACE (CTQ sum score) and total amount of faces misclassified as angry in both presentation time conditions. The displayed graph represents the positive correlation between misclassifications as angry and ACE in patients with BPD (*r* = .43, *p* = .023, Bonferroni-Holm-corrected)

To test our first hypothesis, proposing that patients with BPD would misclassify facial expressions more often as angry compared to healthy volunteers, we analyzed proportion of correct responses and types of errors. Emotion recognition accuracy was high for both patients with BPD (*M* = 93.6%, *SD* = 5.0%) and healthy volunteers (*M* = 93.5%, *SD* = 4.9%) across experimental conditions. Participants were significantly better at recognizing facial expressions in the long than in the brief condition (main effect presentation time, *F* [1, 69] = 42.27, *p* < .001, η^2^ = .38). While no significant effect including the factor group emerged across presentation time conditions (all *F* ≤ 2.29, *p* ≥ .103, η^2^ ≤ .03), separate analyses of the two presentation time conditions revealed a significant group by emotional expression interaction in the brief condition (*F* [3, 207] = 3.07, *p* = .041, η^2^ = .04), but not in the long condition (*F* [3, 207] = 0.75, *p* = .467, η^2^ = .01). In the brief condition, healthy volunteers were significantly more accurate in classifying happy compared to fearful faces (*p* < .01) as well as neutral compared to angry (*p* < .01) and fearful faces (*p* < .01), while this was not shown for patients with BPD. However, pairwise comparisons between patients with BPD and healthy volunteers did not reach statistical significance. Furthermore, patients with BPD and healthy volunteers misclassified facial expressions more often as angry than as happy or as neutral and more often as fearful than as happy (all *p*-values < .01) across presentation time conditions (main effect error type, *F* [3, 207] = 10.75, *p* < .001, η^2^ = .14; see Fig. 1a). No significant group difference was found in terms of types of errors when analyzing the two presentation time conditions together or separately (no significant effect including the factor group, all *F* ≤ .48, *p* ≥ .581, η^2^ ≤ .01). Thus, these results did not confirm our a priori hypothesis.

### Hypothesis 2

To investigate our second hypothesis, stating that patients with BPD would show a visual hypervigilance towards the eyes of angry faces compared to healthy volunteers, we analyzed proportion and latency of initial saccades.

**Fig. 2:**
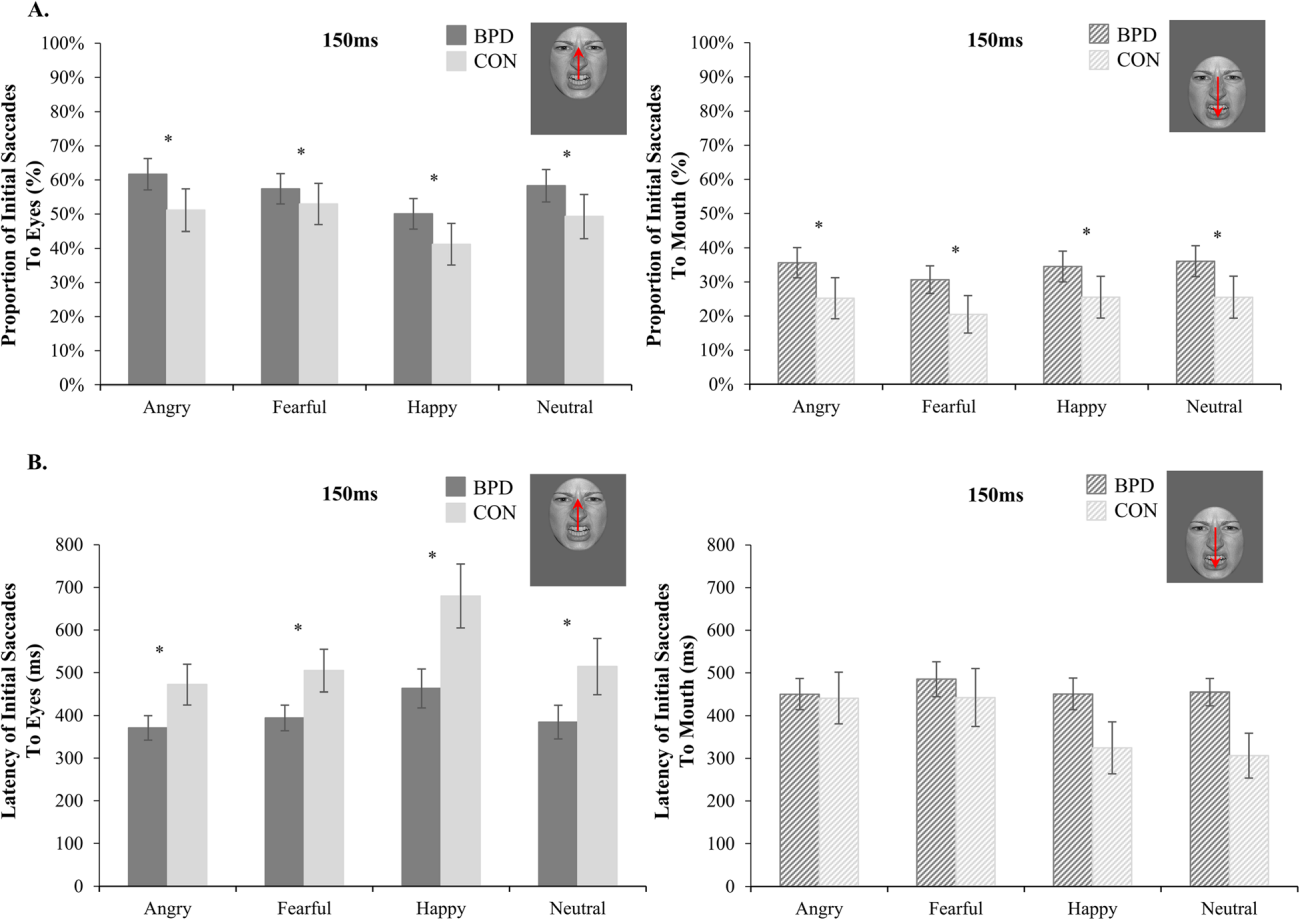
Proportion and latency of initial saccades in patients with BPD and healthy volunteers in the brief condition. **a**. Mean proportion of initial saccades (in %, ± one standard error) to the eyes (left graph) and to the mouth (right graph) in patients with borderline personality disorder (BPD) and healthy volunteers (CON) in the brief condition (150 ms). The displayed graphs represent the significant group by presentation time interaction, with patients with BPD showing more initial saccades compared to healthy volunteers across all experimental conditions in the brief condition. Please note that only significant group differences are highlighted; refer to text and online supplement for further significant effects. * *p* < .05. **b**. Mean latency of initial saccades (in ms, ± one standard error) to the eyes (left graph) and the mouth (right graph) of patients with borderline personality disorder (BPD) and healthy volunteers (CON) in the brief condition (150 ms). The displayed graphs represent the significant group by initial fixation interaction, with patients with BPD showing faster initial saccades towards the eyes compared to healthy volunteers. Please note that only significant group differences are highlighted; refer to text and online supplement for further significant effects. * *p* < .05

Participants generally made more initial saccades in the long than in the brief condition (main effect presentation time, *F* [1, 69] = 122.01, *p* < .001, η^2^ = .64). In addition, a significant group by presentation time interaction emerged (*F* [1, 69] = 6.62, *p* = .012, η^2^ = .09), with patients with BPD showing more initial saccades compared to healthy volunteers in the brief condition (*p* < .05), however, irrespective of emotional expression or initial fixation (see Figure 2a). Separate analyses of the two presentation time conditions did not yield any additional significant group effects.

With regard to latencies of initial saccades, we could analyze only a subsample of *n* = 37 patients with BPD and *n* = 19 healthy volunteers in the long condition and *n* = 22 patients with BPD and *n* = 8 healthy volunteers in the brief condition since the other participants did not show any initial saccade (eye movements exceeding 1° within the time interval of 150 to 1000 ms after facial stimulus onset) towards the other major facial feature in at least one of the eight experimental conditions (initial fixation × emotional expression). Since fixations may rest on the initially fixated major facial feature without any shifts of orientation, and initial saccades may not be required for accurate emotion recognition, these trials may not be classified as invalid [27]. According to a post-hoc sensitivity analysis, the sample size of 30 participants is still large enough to detect medium group by condition interactions (η^2^ = .07) with a statistical power of 1-β ≥ .80 [33]. Overall, participants made faster initial saccades in the long than in the brief condition (main effect presentation time, *F* [1, 26] = 20.99, *p* < .001, η^2^ = .45). Furthermore, a significant group by initial fixation interaction emerged (*F* [1, 26] = 8.16, *p* = .008, η^2^ = .24), with patients with BPD showing faster initial saccades towards the eyes compared to healthy volunteers. This effect was qualified by a significant group by presentation time by initial fixation interaction (*F* [1, 26] = 4.53, *p* = .043, η^2^ = .15; see Fig. 2b). Post-hoc pairwise comparisons within each presentation time condition revealed that patients with BPD exhibited faster initial saccades towards the eyes than towards the mouth in the long condition (*p* < .05) and faster initial saccades towards the eyes compared to healthy volunteers in the brief condition (*p* < .05). In the brief condition, healthy volunteers showed the exact opposite gazing behavior with faster initial saccades towards the mouth than towards the eyes (*p* < .01). Thus, and partly in line with our a priori hypothesis, patients with BPD showed a visual hypervigilance in terms of faster initial saccades towards the eyes of briefly presented emotional and neutral faces in general rather than towards the eyes of angry faces in particular.

### Hypothesis 3

**Fig. 3:**
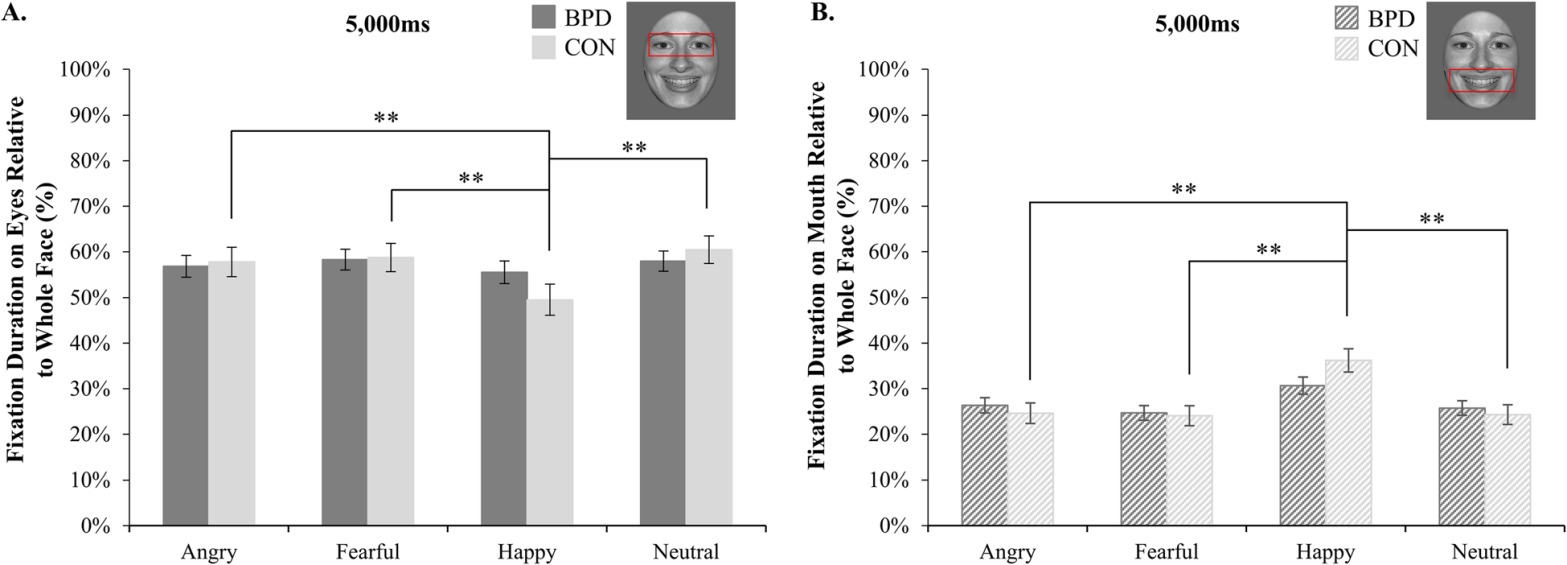
Fixation patterns of patients with BPD and healthy volunteers. Mean fixation duration on the eye (**a**) or the mouth region (**b**) relative to the whole face (in %, ± one standard error) of patients with borderline personality disorder (BPD) and healthy volunteers (CON) in the long condition (5000 ms). The displayed graphs represent the significant group by facial feature by emotional expression interaction, with patients with BPD not modulating their fixation patterns according to the emotion presented like healthy volunteers. Please note that only significant group differences are highlighted; refer to text for further significant effects. ** *p* < .01

To examine our third hypothesis, expecting longer fixation durations on the eye region regardless of emotional valence in patients with BPD compared to healthy volunteers, we analyzed the fixation durations in the long condition. Participants generally fixated the eye region longer than the mouth region (main effect facial feature, *F*[1, 69] = 100.26, *p* < .001, η^2^ = .59) and happy faces longer than angry and fearful faces (main effect emotional expression, *F*[3, 207] = 6.29, *p* < .001, η^2^ = .08). With regard to our a priori hypothesis, a significant facial feature by emotional expression interaction (*F*[3, 207] = 23.13, *p* < .001, η^2^ = .25) and a significant group by facial feature by emotional expression interaction (*F*[3, 207] = 5.65, *p* = .001, η^2^ = .08) were revealed. While healthy volunteers fixated the eyes of angry, fearful, and neutral faces significantly longer than the eyes of happy faces and the mouth of happy faces significantly longer than the mouth of angry, fearful, and neutral faces (all *p*-values < .01), this could not be shown for patients with BPD (see Fig. 3). Contrary to our a priori hypothesis, however, patients with BPD did not fixate the eyes of emotional and neutral faces longer than healthy volunteers.

### Hypothesis 4

To test our fourth hypothesis concerning the positive association between interpersonal threat hypersensitivity and ACE in patients with BPD, we performed correlational analyses with the CTQ sum score in the patient sample only. In patients with BPD, self-reported ACE were positively correlated with misclassifying fearful, happy, and neutral faces as angry (*r* = .43, *p* = .023, Bonferroni-Holm corrected; see Fig. 1b). Due to outliers, we winsorized our data (i.e., replaced the total numbers of misclassifications as angry of five patients with BPD with the next highest number that was not an outlier [48]). By doing this, the association was reduced to a non-significant statistical trend (*r* = .28, *p* = .062). Correlational analyses between ACE and eye-tracking performance in reaction to angry faces did not yield any significant associations (*r* ≤ .38, *p* ≥ .185, Bonferroni-Holm corrected). Please note that the correlational analyses with latencies of initial saccades are only based on a smaller subsample (see Hypothesis 2). Taken together, these results partly confirmed our a priori hypothesis.

### Additional behavioral results

In addition to our hypothesis-driven results and in line with previous studies (e.g., [27, 28, 32]), we were able to replicate a number of more general behavioral and eye-tracking effects of the emotion classification paradigm in patients with BPD and healthy volunteers, which are presented in an online supplement.

## Discussion

To the best of our knowledge, this is the first study to investigate the relationship between behavioral and eye-tracking measures of interpersonal threat sensitivity and ACE in patients with BPD. Our results, although partly inconsistent, point towards a visual hypervigilance for the eye region and a childhood trauma-related anger bias in patients with BPD, potentially supporting the prevalent assumption that interpersonal threat hypersensitivity is indeed associated with early life adversity in BPD.

In line with previous studies (e.g., [27, 28]), both patients with BPD and healthy volunteers were highly accurate in classifying facial expressions across presentation time conditions. Consistent with the notion that patients with BPD do not experience general impairments in emotion recognition but specific subtle deficits in the processing of interpersonal threat cues [3], patients with BPD did not differ from healthy volunteers with regard to the proportion of correctly identified facial expressions. Contrary to our first hypothesis, however, both patients and healthy volunteers misclassified facial expressions more often as angry than as happy or as neutral across presentation time conditions, thus revealing an anger bias which was not specific for patients with BPD. This result was unexpected since several studies document an enhanced tendency to perceive anger in emotional faces in patients with BPD compared to healthy or clinical control groups (e.g., [13–17, 27, 29]), which seems to persist even after symptomatic remission [49] [but see [50], for inconsistent findings]. Differences in the type of stimuli used may be considered as one possible explanation for this divergent finding. With the exception of Bertsch et al. [27, 28], in all of the above-mentioned studies, pictures of morphed instead of full intensity emotional facial expressions were presented. Thereby, the difficulty of the experimental task, the probability of misclassifications and thus, the tendency of more readily identifying signals of interpersonal threat may have been increased.

Our results are only partly in line with our second hypothesis. Patients with BPD showed more initial saccades compared to healthy volunteers in the brief condition, regardless of emotional expression and initial fixation. This enhanced tendency to reflexively direct attention towards diagnostically relevant features of facial expressions could be interpreted as a manifestation of a hypervigilant mindset constantly searching for potential interpersonal threat cues in the environment [51]. Moreover, patients with BPD showed faster initial saccades towards the eyes of briefly presented emotional and neutral expressions. Recognizing threatening facial expressions (i.e., anger) relies mostly on allocating visual attention to the face’s eye region [30, 31]. Hence, directing one’s gaze as quickly as possible towards the facial feature that provides the most salient information about interpersonal threat cues might mirror a highly reflexive hypersensitivity for potential signs of social threat in patients with BPD. Contrary to our second hypothesis, however, the enhanced proportion and latency of initial saccades were not specific for the eyes of angry faces (cf. [28]), but could be found for any facial expression presented. Thus, our findings might also point towards a visual hypervigilance for social cues in general rather than for interpersonal threat cues in particular. In line with Kaiser et al. [29], factors other than interpersonal threat hypersensitivity should be considered to elucidate this visual attention bias for the eye region of emotional faces in general rather than of angry faces in particular. Following Lazarus et al. [52], inconsistent findings concerning facial affect recognition in BPD might be explained by differences in the experimental setup (i.e., fMRI vs. laboratory, set of facial stimuli), influence of psychotropic medications, and co-occurring symptom disorders. With regard to experimental methodology and medication status, a hypervigilance for the eye region was found in medicated and unmedicated patients with BPD presented with ambiguous or full intensity emotional expressions in a previous study by Kaiser et al. [29] and the present study. However, high rates of comorbid axis I disorders, especially a 30% rate of BPD patients with a current diagnosis of social phobia, might account for this generalized hypersensitivity for social stimuli in the present sample. This would be in line with a previous eye-tracking study [32], reporting an enhanced attentional orienting towards the eye region in patients with social phobia as compared to healthy controls, regardless of the type of emotional expression displayed.

Contrary to our third hypothesis, patients with BPD did not fixate the eye region longer than healthy volunteers, irrespective of emotional valence. Hence, our results are inconsistent with a recent eye-tracking study [29], reporting longer fixations on the eye region of ambiguous faces in patients with BPD compared to non-patients. Our findings are, however, in line with a previous eye-tracking study of our group [27], which also failed to detect longer fixation durations on the eye region of emotional expressions displayed in full intensity in a smaller, independent sample of female patients with BPD compared to healthy volunteers. Employing full intensity rather than ambiguous blends of emotional facial expressions might, again, have played a role in producing these heterogeneous findings across studies. Nevertheless, patients with BPD did exhibit aberrant fixation patterns compared to healthy volunteers. While healthy volunteers fixated longer on the facial feature more relevant for the recognition of the distinct emotional expression presented, patients with BPD did not adapt their fixation patterns accordingly.

In line with our fourth hypothesis, the results of the correlational analyses revealed more misclassifications of facial expressions as angry in patients with more self-reported ACE. This result should, however, be interpreted cautiously. Due to high emotion recognition accuracy in patients with BPD, the total number of anger misclassifications lacks sufficient variance and our correlational analysis might be biased by outliers. Nonetheless, calculating the correlation with winsorized misclassifications as angry still revealed a positive, medium-sized association which can be characterized as a non-significant statistical trend (*p* < .10). Together with previous studies [23–25, 53], the current findings may provide evidence supporting the assumption that interpersonal threat hypersensitivity is indeed associated with early life adversity in patients with BPD [but see [17], for inconsistent results]. Contrary to our expectations, however, we did not find significant associations between eye-tracking measures of interpersonal threat hypersensitivity and ACE.

Our study has some strengths, investigating behavioral and eye-tracking measures of interpersonal threat sensitivity and their association with ACE in well-matched, unmedicated samples of patients with BPD and healthy volunteers. Several limitations should, however, be acknowledged. First, we included only female participants. Since sex differences in visual attention allocation during facial emotion recognition have been documented [54], we cannot draw conclusions concerning patients with BPD of other sex than female. Second, our patient sample reported high rates of psychiatric comorbidities. While high rates of axis I comorbidities are a common finding in the literature [55] and thus emphasize the representativeness of our sample, it also questions the specificity of our results for BPD, especially since our study lacks a clinical control group. The absence of a clinical control group therefore precludes any inferences regarding the BPD-specific nature of our findings. Third, since our healthy control group reported only limited exposure to ACE (CTQ sum score), we focused the correlational analyses with early life adversity on the patient group only. Of note, exploratory correlational analyses between ACE and behavioral and eye-tracking measures of interpersonal threat sensitivity in the healthy control group did not yield any significant associations (*r* ≤ .41, *p* ≥ .691, Bonferroni-Holm corrected). Due to insufficient power and lack of variance in CTQ scores, however, we cannot draw any conclusions from these non-significant findings. It therefore remains unclear whether the association between interpersonal threat hypersensitivity and ACE is due to the diagnosis of BPD or due to early life maltreatment in general. Future studies should include BPD patient, healthy and clinical control groups, each with male and female participants with a broad range of ACE, to get a better insight in the specificity of this association. Fourth, although our sample size was large enough to detect small to medium group by condition interactions for all behavioral and eye-tracking measures of interpersonal threat hypersensitivity as well as medium to large correlations between these measures of interpersonal threat hypersensitivity and ACE, it was not adequate to detect small to medium correlations. This applies especially to the analyses of saccadic latencies which had to be conducted with a smaller subsample of patients. Given the lack of power, expected associations between ACE and eye-tracking measures of interpersonal threat hypersensitivity in general, and latencies of initial saccades in particular, might have not been detected and further replication studies with larger groups of patients with BPD are needed to allow for strong conclusions about non-significant effects. Fifth, we presented full intensity static facial stimuli that do not adequately mirror the dynamics and subtlety of emotional expressions in our everyday social interactions. Employing recently developed dynamic sets of non-stereotypical facial expressions [56, 57]) in future studies may extend our findings to more naturalistic settings. Moreover, it might render the experimental task more difficult and thus lead to a higher variance in emotion recognition accuracy and types of misclassifications. Sixth, we applied a matching approach to “control for” potential differences in sex, age, and intelligence between the patient and healthy control group. According to classic papers by Meehl [58], and Miller and Chapman [59], by doing so, we might have generated a systematic mismatch in other variables and thus introduced artifacts in our data. Finally, due to the cross-sectional design of our study, causal inferences cannot be drawn from our findings. This is of particular importance with regard to the correlational analyses, given that retrospective, subjective self-reports of ACE are prone to various memory biases [60] and cannot be interpreted interchangeably with prospective measures of ACE [61].

## Conclusions

In summary, our current study suggests an association between interpersonal threat hypersensitivity in terms of an anger bias in BPD and ACE. Preliminary models postulate that the hypervigilance towards potentially threatening interpersonal cues might lead to severe impairments in psychosocial functioning of patients with BPD [62]. Effective and efficient treatment strategies to reduce the attentional bias for potential signs of social threat are therefore needed. In BPD, increased amygdala activation to (negative) emotional stimuli has been suggested as a neural underpinning of interpersonal threat hypersensitivity [3] and deficient amygdala habituation to threatening cues has been shown to be associated with ACE [63]. In a recent single-arm trial, patients with BPD succeeded in downregulating their amygdala activation and reported less BPD symptoms after receiving four sessions of amygdala neurofeedback [64]. Together with psychotherapeutic interventions to reduce threat-related attentional biases (cf. [65]), these efforts might lead to novel treatment approaches to reduce threat hypersensitivity and interpersonal dysfunction in patients with BPD and ACE.

## Supplementary Information

**Additional file 1.** Supplemental_Material The supplemental material contains more extensive detail on our methods (psychometric properties of the questionnaires used in our study, stimulus material, and analysis of eye-tracking data) and our results (replication of general behavioral and eye-tracking effects of the emotion classification paradigm).

## Data Availability

The datasets used and analyzed during the current study are available from the corresponding author on reasonable request.

## Abbreviations

ACE: Adverse childhood experiences
BPD: Borderline personality disorder

## References

[1] Crowell SE, Beauchaine TP, Linehan MM. A biosocial developmental model of borderline personality: elaborating and extending Linehan's theory. Psychol Bull. 2009. 10.1037/a0015616. 10.1037/a0015616 PMC2696274 19379027

[2] Carpenter RW, Trull TJ. Components of emotion dysregulation in borderline personality disorder: a review. Curr Psychiatry Rep. 2013. 10.1007/s11920-012-0335-2. 10.1007/s11920-012-0335-2 PMC3973423 23250816

[3] Bertsch K, Hillmann K, Herpertz SC. Behavioral and neurobiological correlates of disturbed emotion processing in borderline personality disorder. Psychopathology. 2018. 10.1159/000487363. 10.1159/000487363 29539627

[4] Mancke F, Herpertz SC, Bertsch K. Aggression in borderline personality disorder: a multidimensional model. Personal Disord. 2015. 10.1037/per0000098. 10.1037/per0000098 26191822

[5] Daros AR, Zakzanis KK, Ruocco AC. Facial emotion recognition in borderline personality disorder. Psychol Med. 2013. 10.1017/s0033291712002607. 10.1017/S0033291712002607 23149223

[6] Domes G, Schulze L, Herpertz SC. Emotion recognition in borderline personality disorder - a review of the literature. J Personal Disord. 2009. 10.1521/pedi.2009.23.1.6. 10.1521/pedi.2009.23.1.6 19267658

[7] Mitchell AE, Dickens GL, Picchioni MM. Facial emotion processing in borderline personality disorder: a systematic review and meta-analysis. Neuropsychol Rev. 2014. 10.1007/s11065-014-9254-9. 10.1007/s11065-014-9254-9 24574071

[8] Matzke B, Herpertz SC, Berger C, Fleischer M, Domes G. Facial reactions during emotion recognition in borderline personality disorder: a facial electromyography study. Psychopathology. 2014. 10.1159/000351122. 10.1159/000351122 24021701

[9] Mier D, Lis S, Esslinger C, Sauer C, Hagenhoff M, Ulferts J, et al. Neuronal correlates of social cognition in borderline personality disorder. Soc Cogn Affect Neurosci. 2013. 10.1093/scan/nss028. 10.1093/scan/nss028 PMC3682436 22362841

[10] Catalan A, Gonzalez de Artaza M, Bustamante S, Orgaz P, Osa L, Angosto V, et al. Differences in facial emotion recognition between first episode psychosis, borderline personality disorder and healthy controls. PLoS One. 2016. 10.1371/journal.pone.0160056. 10.1371/journal.pone.0160056 PMC4965014 27467692

[11] Daros AR, Uliaszek AA, Ruocco AC. Perceptual biases in facial emotion recognition in borderline personality disorder. Personal Disord. 2014. 10.1037/per0000056. 10.1037/per0000056 24588064

[12] Fenske S, Lis S, Liebke L, Niedtfeld I, Kirsch P, Mier D. Emotion recognition in borderline personality disorder: effects of emotional information on negative bias. Borderline Personal Disord Emot Dysregulation. 2015. 10.1186/s40479-015-0031-z. 10.1186/s40479-015-0031-z PMC4579484 26401312

[13] Berenson KR, Dochat C, Martin CG, Yang X, Rafaeli E, Downey G. Identification of mental states and interpersonal functioning in borderline personality disorder. Personal Disord. 2018. 10.1037/per0000228. 10.1037/per0000228 PMC5425332 27831693

[14] Ferreira G, Sanches R, Crippa J, Mello M, Osório F. Borderline personality disorder and bias in the recognition of facial expressions of emotion: a pathway to understand the psychopathology. Arch Clin Psychiatry. 2018. 10.1590/0101-60830000000146.

[15] Domes G, Czieschnek D, Weidler F, Berger C, Fast K, Herpertz SC. Recognition of facial affect in borderline personality disorder. J Personal Disord. 2008. 10.1521/pedi.2008.22.2.135. 10.1521/pedi.2008.22.2.135 18419234

[16] Izurieta Hidalgo NA, Oelkers-Ax R, Nagy K, Mancke F, Bohus M, Herpertz SC, et al. Time course of facial emotion processing in women with borderline personality disorder: an ERP study. J Psychiatry Neurosci. 2016. 10.1503/jpn.140215. 10.1503/jpn.140215 PMC4688024 26269211

[17] Veague HB, Hooley JM. Enhanced sensitivity and response bias for male anger in women with borderline personality disorder. Psychiatry Res. 2014. 10.1016/j.psychres.2013.12.045. 10.1016/j.psychres.2013.12.045 24485062

[18] Thome J, Liebke L, Bungert M, Schmahl C, Domes G, Bohus M, et al. Confidence in facial emotion recognition in borderline personality disorder. Personal Disord. 2016. 10.1037/per0000142. 10.1037/per0000142 26389624

[19] van Dijke A, van’ t Wout M, Ford JD, Aleman A. Deficits in degraded facial affect labeling in schizophrenia and borderline personality disorder. PLoS One. 2016. 10.1371/journal.pone.0154145. 10.1371/journal.pone.0154145 PMC4907495 27300727

[20] de Aquino Ferreira LF, Queiroz Pereira FH, Neri Benevides AML, Aguiar Melo MC. Borderline personality disorder and sexual abuse: a systematic review. Psychiatry Res. 2018. 10.1016/j.psychres.2018.01.043. 10.1016/j.psychres.2018.01.043 29407572

[21] Zanarini MC, Williams AA, Lewis RE, Reich RB, Vera SC, Marino MF, et al. Reported pathological childhood experiences associated with the development of borderline personality disorder. Am J Psychiatry. 1997. 10.1176/ajp.154.8.1101. 10.1176/ajp.154.8.1101 9247396

[22] Linehan M (1993). Cognitive–behavioral treatment of borderline personality disorder.

[23] Nicol K, Pope M, Sprengelmeyer R, Young AW, Hall J. Social judgement in borderline personality disorder. PLoS One. 2013. 10.1371/journal.pone.0073440. 10.1371/journal.pone.0073440 PMC3819347 24223110

[24] Kaiser D, Jacob GA, van Zutphen L, Siep N, Sprenger A, Tuschen-Caffier B, et al. Patients with borderline personality disorder and comorbid PTSD show biased attention for threat in the facial dot-probe task. J Behav Ther Exp Psychiatry. 2020. 10.1016/j.jbtep.2018.11.005. 10.1016/j.jbtep.2018.11.005 30563688

[25] Bruene M, Ebert A, Kolb M, Tas C, Edel MA, Roser P. Oxytocin influences avoidant reactions to social threat in adults with borderline personality disorder. Hum Psychopharmacol. 2013. 10.1002/hup.2343. 10.1002/hup.2343 23950057

[26] Lowyck B, Luyten P, Vanwalleghem D, Vermote R, Mayes LC, Crowley MJ. What's in a face? Mentalizing in borderline personality disorder based on dynamically changing facial expressions. Personal Disord. 2016. 10.1037/per0000144. 10.1037/per0000144 26461044

[27] Bertsch K, Krauch M, Stopfer K, Haeussler K, Herpertz SC, Gamer M. Interpersonal threat sensitivity in borderline personality disorder: an eye-tracking study. J Personal Disord. 2017. 10.1521/pedi_2017_31_273. 10.1521/pedi_2017_31_273 28072041

[28] Bertsch K, Gamer M, Schmidt B, Schmidinger I, Walther S, Kästel T, et al. Oxytocin and reduction of social threat hypersensitivity in women with borderline personality disorder. Am J Psychiatry. 2013. 10.1176/appi.ajp.2013.13020263. 10.1176/appi.ajp.2013.13020263 23982273

[29] Kaiser D, Jacob GA, van Zutphen L, Siep N, Sprenger A, Tuschen-Caffier B, et al. Biased attention to facial expressions of ambiguous emotions in borderline personality disorder: an eye-tracking study. J Personal Disord. 2019. 10.1521/pedi_2019_33_363. 10.1521/pedi_2019_33_363 30689505

[30] Schurgin MW, Nelson J, Iida S, Ohira H, Chiao JY, Franconeri SL. Eye movements during emotion recognition in faces. J Vis. 2014. 10.1167/14.13.14. 10.1167/14.13.14 25406159

[31] Calvo MG, Fernandez-Martin A, Gutierrez-Garcia A, Lundqvist D. Selective eye fixations on diagnostic face regions of dynamic emotional expressions: KDEF-dyn database. Sci Rep. 2018. 10.1038/s41598-018-35259-w. 10.1038/s41598-018-35259-w PMC6242984 30451919

[32] Boll S, Bartholomaeus M, Peter U, Lupke U, Gamer M. Attentional mechanisms of social perception are biased in social phobia. J Anxiety Disord. 2016. 10.1016/j.janxdis.2016.04.004. 10.1016/j.janxdis.2016.04.004 PMC4877390 27131909

[33] Faul F, Erdfelder E, Lang AG, Buchner A. G*power 3: a flexible statistical power analysis program for the social, behavioral, and biomedical sciences. Behav Res Methods. 2007. 10.3758/bf03193146. 10.3758/bf03193146 17695343

[34] Bodenschatz CM, Skopinceva M, Russ T, Suslow T. Attentional bias and childhood maltreatment in clinical depression - an eye-tracking study. J Psychiatr Res. 2019. 10.1016/j.jpsychires.2019.02.025. 10.1016/j.jpsychires.2019.02.025 30870713

[35] Bernstein DP, Fink L (1998). Childhood Trauma Questionnaire: a retrospective self-report. Manual.

[36] Schmahl C, Herpertz SC, Bertsch K, Ende G, Flor F, Kirsch P, et al. Mechanisms of disturbed emotion processing and social interaction in borderline personality disorder: state of knowledge and research agenda of the German clinical research unit. Borderline Personal Disord Emot Dysregulation. 2014. 10.1186/2051-6673-1-12. 10.1186/2051-6673-1-12 PMC4579501 26401296

[37] First MB, Spitzer RL, Gibbon M, Williams JBW (1995). Structured clinical interview for DSM-IV (SCID-I).

[38] Loranger AW, Sartorius N, Andreoli A, Berger P, Buchheim P, Channabasavanna SM, et al. The international personality disorder examination (IPDE): the World Health Organization/alcohol, drug abuse, and mental health administration international pilot study of personality disorders. Arch Gen Psychiatry. 1994. 10.1001/archpsyc.1994.03950030051005. 10.1001/archpsyc.1994.03950030051005 8122958

[39] Bohus M, Kleindienst N, Limberger MF, Stieglitz RD, Domsalla M, Chapman AL, et al. The short version of the borderline symptom list (BSL-23): development and initial data on psychometric properties. Psychopathology. 2009. 10.1159/000173701. 10.1159/000173701 19023232

[40] Beck AT, Steer RA, Brown GK (1996). Manual for the Beck depression inventory-II.

[41] Laux L, Glanzmann P, Schaffner P, Spielberger CD (1981). Das state-trait-Angstinventar (STAI).

[42] Gratz K, Roemer L. Multidimensional assessment of emotion regulation and dysregulation: development, factor structure, and initial validation of the difficulties in emotion regulation scale. J Psychopathol Behav Assess. 2004. 10.1007/s10862-008-9102-4.

[43] Heller KA, Kratzmeier H, Lengfelder A (1998). Matrizen-test-manual, band 2. Ein Handbuch mit deutschen Normen zu den advanced progressive matrices von J. C. Raven [matrix test manual. A handbook for Raven’s advanced progressive matrices].

[44] Scheller E, Büchel C, Gamer M. Diagnostic features of emotional expressions are processed preferentially. PLoS One. 2012. 10.1371/journal.pone.0041792. 10.1371/journal.pone.0041792 PMC3405011 22848607

[45] Boll S, Gamer M. 5-HTTLPR modulates the recognition accuracy and exploration of emotional facial expressions. Front Behav Neurosci. 2014. 10.3389/fnbeh.2014.00255. 10.3389/fnbeh.2014.00255 PMC4107864 25100964

[46] Huynh H, Feldt LS. Estimation of the box correction for degrees of freedom from sample data in randomized block and split-plot designs. J Educ Behav Stat. 1976. 10.3102/10769986001001069.

[47] Holm S (1979). A simple sequentially rejective multiple test procedure. Scand Stat Theory Appl.

[48] Dixon WJ (1960). Simplified estimation from censored normal samples. Ann Math Stat.

[49] Kleindienst N, Hauschild S, Liebke L, Thome J, Bertsch K, Hensel S, et al. A negative bias in decoding positive social cues characterizes emotion processing in patients with symptom-remitted borderline personality disorder. Borderline Personal Disord Emot Dysregulation. 2019. 10.1186/s40479-019-0114-3. 10.1186/s40479-019-0114-3 PMC6858731 31788316

[50] Schneider I, Bertsch K, Izurieta Hidalgo NA, Muller LE, Schmahl C, Herpertz SC. Remnants and changes in facial emotion processing in women with remitted borderline personality disorder: an EEG study. Eur Arch Psychiatry Clin Neurosci. 2018. 10.1007/s00406-017-0841-7. 10.1007/s00406-017-0841-7 28956145

[51] Kimble M, Boxwala M, Bean W, Maletsky K, Halper J, Spollen K, et al. The impact of hypervigilance: evidence for a forward feedback loop. J Anxiety Disord. 2014. 10.1016/j.janxdis.2013.12.006. 10.1016/j.janxdis.2013.12.006 PMC4211931 24507631

[52] Lazarus SA, Cheavens JS, Festa F, Zachary RM. Interpersonal functioning in borderline personality disorder: a systematic review of behavioral and laboratory-based assessments. Clin Psychol Rev. 2014. 10.1016/j.cpr.2014.01.007. 10.1016/j.cpr.2014.01.007 24534643

[53] Wagner AW, Linehan MM. Facial expression recognition ability among women with borderline personality disorder: implications for emotion regulation? J Personal Disord. 1999. 10.1521/pedi.1999.13.4.329. 10.1521/pedi.1999.13.4.329 10633314

[54] Hall JK, Hutton SB, Morgan MJ. Sex differences in scanning faces: does attention to the eyes explain female superiority in facial expression recognition? Cognit Emot. 2010. 10.1080/02699930902906882.

[55] Shah R, Zanarini MC. Comorbidity of borderline personality disorder: current status and future directions. Psychiatr Clin North Am. 2018. 10.1016/j.psc.2018.07.009. 10.1016/j.psc.2018.07.009 30447726

[56] Calvo MG, Fernandez-Martin A, Recio G, Lundqvist D. Human observers and automated assessment of dynamic emotional facial expressions: KDEF-dyn database validation. Front Psychol. 2018. 10.3389/fpsyg.2018.02052. 10.3389/fpsyg.2018.02052 PMC6212581 30416473

[57] Yitzhak N, Giladi N, Gurevich T, Messinger DS, Prince EB, Martin K, et al. Gently does it: humans outperform a software classifier in recognizing subtle, nonstereotypical facial expressions. Emotion. 2017. 10.1037/emo0000287. 10.1037/emo0000287 28406679

[58] Meehl PE. High school yearbooks: a reply to Schwarz. J Abnorm Psychol. 1971. 10.1037/h0030750. 10.1037/h0031999 5156439

[59] Miller GA, Chapman JP. Misunderstanding analysis of covariance. J Abnorm Psychol. 2001. 10.1037/0021-843X.110.1.40. 10.1037//0021-843x.110.1.40 11261398

[60] Baldwin JR, Reuben A, Newbury JB, Danese A. Agreement between prospective and retrospective measures of childhood maltreatment: a systematic review and meta-analysis. JAMA Psychiatry. 2019. 10.1001/jamapsychiatry.2019.0097. 10.1001/jamapsychiatry.2019.0097 PMC6551848 30892562

[61] Danese A. Annual research review: rethinking childhood trauma-new research directions for measurement, study design and analytical strategies. J Child Psychol Psychiatry. 2020. 10.1111/jcpp.13160. 10.1111/jcpp.13160 31762042

[62] Herpertz SC, Jeung H, Mancke F, Bertsch K. Social dysfunctioning and brain in borderline personality disorder. Psychopathology. 2014. 10.1159/000365106. 10.1159/000365106 25378381

[63] Bilek E, Itz ML, Stößel G, Ma R, Berhe O, Clement L, et al. Deficient amygdala habituation to threatening stimuli in borderline personality disorder relates to adverse childhood experiences. Biol Psychiatry. 2019. 10.1016/j.biopsych.2019.06.008. 10.1016/j.biopsych.2019.06.008 31366446

[64] Zähringer J, Ende G, Santangelo P, Kleindienst N, Ruf M, Bertsch K, et al. Improved emotion regulation after neurofeedback: a single-arm trial in patients with borderline personality disorder. NeuroImage Clin. 2019. 10.1016/j.nicl.2019.102032. 10.1016/j.nicl.2019.102032 PMC6978219 31795041

[65] Vazquez C, Duque A, Blanco I, Pascual T, Poyato N, Lopez-Gomez I, et al. CBT and positive psychology interventions for clinical depression promote healthy attentional biases: an eye-tracking study. Depress Anxiety. 2018. 10.1002/da.22786. 10.1002/da.22786 30028564

