# Supplementary material for: An eye-tracking study of interpersonal threat sensitivity and adverse childhood experiences in borderline personality disorder
Source: Borderline Personal Disord Emot Dysregul. 2021 Jan 4;8:2. doi: 10.1186/s40479-020-00141-7 (PMC7784013; doi:10.1186/s40479-020-00141-7)
Supplement: Supplementary file 1 — Additional file 1. Supplemental_Material The supplemental material contains more extensive detail on our methods (psychometric properties of the questionnaires used in our study, stimulus material, and analysis of eye-tracking data) and our results (replication of general behavioral and eye-tracking effects of the emotion classification paradigm). [file 40479_2020_141_MOESM1_ESM.docx]

**Supplemental Material**

**Methods**

**Psychometric properties of self-report questionnaires and intelligence measure**

Adverse childhood experiences (ACE) were measured with the Childhood Trauma Questionnaire [CTQ; 1]. In our sample, the internal consistency of four of the five subscales ranged from good (α = .87 for physical abuse) to excellent (α = .98 for sexual abuse), with the exception of α = .68 for physical neglect (reported internal consistency for the five subscales ranging from α = .55 [physical neglect] to .89 [sexual abuse] and test-retest reliability for the total scale of r_tt_ = .88) [2, 3]. Additionally, we assessed the following clinical aspects to characterize our sample further: BPD symptom severity was measured using the short version of the Borderline Symptom List [BSL-23; 4], a 23-item self-report scale with an excellent internal consistency of α = .97 in our sample (reported internal consistency of α = .94–.97 and test-retest reliability of r_tt_ = .82) [5]. The current degree of depression was assessed with the revised version of Beck’s Depression Inventory [BDI-II; 6], yielding an excellent internal consistency of α = .94 in the current sample (reported internal consistency of α = .84–.94 and test-retest reliability of r_tt_ = .78) [7]. Trait anxiety was measured with the 21-item trait anxiety scale of the State-Trait Anxiety Inventory [STAI; 8] with an excellent internal consistency of α = .97 in our sample (reported internal consistency of α = .90 and test-retest reliability of r_tt_ = .88) [8]. Clinically relevant difficulties in multiple domains of emotion regulation (i.e., lack of emotional awareness, lack of emotional clarity, non-acceptance of negative emotional responses, lack of functional emotion regulation strategies, difficulties in engaging in goal-directed behavior and impulse control issues) were identified using the 36-item Difficulties in Emotion Regulation Scale [DERS; 9] with an excellent internal consistency of α = .96 in the current sample (reported internal consistency of α = .93 and test-retest reliability of r_tt_ = .88) [9]. Finally, intelligence was measured as a matching variable using Raven’s Standard Progressive Matrices [10], requiring the participants to complete 60 matrices and thereby assessing their nonverbal deductive abilities. Internal consistency has been found to be acceptable to excellent across studies (α = .77–.96) and test-retest reliability has been shown to be excellent (r_tt_ = .77–.96) [10].

**Stimulus material**

Photographs of male and female actors, each unambiguously displaying angry, fearful, happy, and neutral expressions, were drawn from the following established picture sets: the Karolinska Directed Emotional Faces [KDEF; 11], the NimStim Face Stimulus Set (http://www.macbrain.org/resources.htm), Pictures of facial affect [12], and the FACES database [13]. The selected photographs were transformed into grayscale images and cumulative brightness was normalized across all pictures. The faces were cropped with an elliptic mask to remove hair and ears and slightly rotated to align both pupils at the same height.

**Analysis of eye-tracking data**

For all eye-tracking measures, we first identified valid trials by eliminating trials containing blinks and fixation changes with an amplitude of more than 1° during a period of -300 to 150ms relative to facial stimulus onset. In our sample, the average proportion of valid trials was 76% (*SD* = 14%), and no significant group difference emerged (patients with BPD: *M* = 74%, *SD* = 14%; healthy volunteers: *M* = 79%, *SD* = 12%, *t*(69) = -1.52, *p* =.134). To remove drifts, we subtracted the baseline prior to the onset of the facial stimulus from the position data of each valid trial.

We calculated the proportion of initial saccades by detecting the first saccades exceeding 1° within the time interval of 150 to 1,000ms after facial stimulus onset. These initial saccades were classified according to whether they were directed towards the other major facial feature. Hence, when the eyes were presented at the former position of the fixation cross, the downward fixation changes towards the mouth were scored, whereas when the mouth was shown at fixation, the upward fixation changes towards the eyes were scored. We then divided these scores by the total number of valid trials in the corresponding experimental condition, yielding proportional values of initial saccades as a function of the experimental manipulation.

We defined latency of initial saccades as the time elapsed from facial stimulus to saccade onset. In line with the calculation of the proportion of initial saccades, for the calculation of the latency of initial saccades we also only considered saccades that were directed towards the other major facial feature presented in the visual periphery (i.e., saccades towards the eyes when the mouth was initially fixated and saccades towards the mouth when the eyes were initially fixated). Latencies were averaged across trials within each experimental condition.

Additionally, we analyzed fixation durations, defined as the amount of time participants spent looking at either the eye or the mouth region (i.e., facial feature) in the long presentation time condition. To this aim, we assessed the cumulative fixation time on predefined rectangular regions of interest, centered on the respective facial feature. Subsequently, we divided the cumulative fixation time on these features by the amount of time the participants spent looking at the presented facial stimulus in general.

**Results**

**Replication of general behavioral and eye-tracking effects**

With regard to proportion of correct responses, participants were significantly better when faces were presented with initial fixation on the eyes than on the mouth (main effect initial fixation, *F*[1, 69] = 13.71, *p* < .001, η^2^ = .17) and at recognizing happy than angry (*p* < .01), fearful (*p* < .01), and neutral faces (*p* < .05) (main effect emotional expression, *F*[3, 207] = 12.04, *p* < .001, η^2^ = .15) across presentation time conditions. They made significantly more errors when identifying angry faces with initial fixation on the mouth compared with fearful and neutral faces, as well as angry faces with initial fixation on the eyes (all *p*-values < .01) (emotional expression by initial fixation interaction, *F*[3, 207] = 5.89, *p* = .002, η^2^ = .08). Moreover, they were significantly worse at recognizing angry and fearful faces in the brief than in the long condition (all *p*-values < .01) (presentation time by emotional expression interaction, *F*[3, 207] = 7.41, *p* = .001, η^2^ = .10).

With regard to response latencies, patients with BPD reacted significantly slower than healthy volunteers across presentation time conditions (main effect group, *F*[1, 69] = 6.51, *p* = .013, η^2^ = .09). Participants responded significantly faster in the brief than in the long condition (main effect presentation time, *F*[1, 69] = 6.31, *p* = .014, η^2^ = .08). Across presentation time conditions, response latencies were significantly shorter when participants identified happy faces compared to all other emotions, and neutral faces compared to angry and fearful faces (all *p*-values < .01) (main effect emotional expression, *F*[3, 207] = 61.61, *p* < .001, η^2^ = .47). The latter effect was qualified by a significant initial fixation by emotional expression interaction (*F*[3, 207] = 6.10, *p* = .001, η^2^ = .08), with participants reacting faster to angry faces with initial fixation on the eyes than on the mouth (*p* < .01). Moreover, a significant presentation time by emotional expression interaction (*F*[3, 207] = 4.71, *p* = .011, η^2^ = .06) emerged, with participants showing significantly faster responses to neutral compared to fearful faces in the brief than in the long condition (*p* < .01).

With regard to proportion of initial saccades, participants generally made more initial saccades towards the eyes than towards the mouth (main effect initial fixation, *F*[1, 69] = 45.71, *p* < .001, η^2^ = .40). Additionally, participants made more initial saccades towards the mouth of happy compared to fearful faces (*p* < .01) and towards the eyes of angry (*p* < .05), fearful (*p* < .01) and neutral faces (*p* < .01) compared to happy faces (initial fixation by emotional expression interaction, *F*[3, 207] = 8.12, *p* < .001, η^2^ = .11).

With regard to latency of initial saccades, participants made faster initial saccades towards angry (*p* < .05) and neutral (*p* < .01) faces compared to happy faces and towards neutral compared to fearful faces (*p* < .05) (main effect emotional expression, *F*[3, 78] = 2.81, *p* = .045, η^2^ = .10). The latter effect was qualified by a significant initial fixation by emotional expression interaction (*F*[3, 78] = 9.23, *p* < .001, η^2^ = .26) and a significant presentation time by initial fixation by emotional expression interaction (*F*[3, 78] = 5.02, *p* = .004, η^2^ = .16). Separate analyses of the two presentation time conditions revealed that participants made faster initial saccades towards the eyes of neutral compared to happy faces and towards the eyes than towards the mouth of fearful and neutral faces in the long condition (all *p*-values < .01). Moreover, participants made faster initial saccades towards the eyes of angry, fearful, and neutral faces compared to happy faces and faster initial saccades towards the mouth than towards the eyes of happy faces in the brief condition (all *p*-values < .01).

**References Supplemental Material**

1. Bernstein DP, Fink L. Childhood Trauma Questionnaire: a retrospective self-report. Manual. San Antonio, TX.: The Psychological Corporation; 1998.

2. Bernstein DP, Fink L, Handelsman L, Foote J, Lovejoy M, Wenzel K, et al. Initial reliability and validity of a new retrospective measure of child abuse and neglect. Am J Psychiatry. 1994; doi:10.1176/ajp.151.8.1132.

3. Klinitzke G, Romppel M, Häuser W, Brähler E, Glaesmer H. The German version of the Childhood Trauma Questionnaire (CTQ): psychometric characteristics in a representative sample of the general population. Psychother Psych Med. 2012; doi:10.1055/s-0031-1295495.

4. Bohus M, Kleindienst N, Limberger MF, Stieglitz RD, Domsalla M, Chapman AL, et al. The short version of the Borderline Symptom List (BSL-23): development and initial data on psychometric properties. Psychopathology. 2009; doi:10.1159/000173701.

5. Wolf M, Limberger MF, Kleindienst N, Stieglitz RD, Domsalla M, Philipsen A, et al. Kurzversion der Borderline-Symptom-Liste (BSL-23): Entwicklung und Überprüfung der psychometrischen Eigenschaften. [Short version of the borderline symptom list (BSL-23): Development and psychometric evaluation]. Psychother Psych Med. 2009; doi:10.1055/s-0028-1104598.

6. Beck AT, Steer RA, Brown GK. Manual for the Beck Depression Inventory-II. San Antonio, TX: The Psychological Corporation; 1996.

7. Kühner C, Bürger C, Keller F, Hautzinger M. Reliability and validity of the revised Beck Depression Inventory (BDI-II). Results from German samples. Nervenarzt. 2007; doi:10.1007/s00115-006-2098-7.

8. Laux L, Glanzmann P, Schaffner P, Spielberger CD. Das State-Trait-Angstinventar (STAI). Göttingen: Beltz; 1981.

9. Gratz K, Roemer L. Multidimensional assessment of emotion regulation and dysregulation: development, factor structure, and initial validation of the difficulties in emotion regulation scale. J Psychopathol Behav Assess. 2004; doi:10.1007/s10862-008-9102-4.

10. Heller KA, Kratzmeier H, Lengfelder A. Matrizen-Test-Manual, Band 2. Ein Handbuch mit deutschen Normen zu den Advanced Progressive Matrices von J. C. Raven [Matrix test manual. A handbook for Raven’s Advanced Progressive Matrices]. Göttingen, Germany: Beltz; 1998.

11. Lundqvist D, Flykt A, Öhman A. The Karolinska Directed Emotional Faces - KDEF (CD ROM). Stockholm: Karolinska Institute, Department of Clinical Neuroscience, Psychology Section; 1998.

12. Ekman P, Friesen WV. Pictures of Facial Affect. Palo Alto, CA: Consulting Psychologists Press; 1976.

13. Ebner NC, Riediger M, Lindenberger U. FACES - a database of facial expressions in young, middle-aged, and older women and men: development and validation. Behav Res Methods. 2010; doi:10.3758/brm.42.1.351.
